# Transposons Hidden in *Arabidopsis thaliana* Genome Assembly Gaps and Mobilization of Non-Autonomous LTR Retrotransposons Unravelled by Nanotei Pipeline

**DOI:** 10.3390/plants10122681

**Published:** 2021-12-06

**Authors:** Ilya Kirov, Pavel Merkulov, Maxim Dudnikov, Ekaterina Polkhovskaya, Roman A. Komakhin, Zakhar Konstantinov, Sofya Gvaramiya, Aleksey Ermolaev, Natalya Kudryavtseva, Marina Gilyok, Mikhail G. Divashuk, Gennady I. Karlov, Alexander Soloviev

**Affiliations:** 1 All-Russia Research Institute of Agricultural Biotechnology, Timiryazevskaya Str. 42, 127550 Moscow, Russia; paulmerkulov97@gmail.com (P.M.); max.dudnikov.07@gmail.com (M.D.); eynzeynkreyn@gmail.com (E.P.); komakhin@gmail.com (R.A.K.); zakhar.konstantinov@mail.ru (Z.K.); sofia.gvaramia@gmail.com (S.G.); mariobrok69@gmail.com (M.G.); divashuk@gmail.com (M.G.D.); karlovg@gmail.com (G.I.K.); A.Soloviev70@gmail.com (A.S.); 2 Kurchatov Genomics Center of ARRIAB, All-Russia Research Institute of Agricultural Biotechnology, Timiryazevskaya Str. 42, 127550 Moscow, Russia; 3 Center of Molecular Biotechnology, Russian State Agrarian University-Moscow Timiryazev Agricultural Academy, 127550 Moscow, Russia; ermol-2012@yandex.ru (A.E.); natalia_kudryavtseva@outlook.com (N.K.)

**Keywords:** transposon insertions, long read sequencing, structural variants, GAG, *ddm1*

## Abstract

Long-read data is a great tool to discover new active transposable elements (TEs). However, no ready-to-use tools were available to gather this information from low coverage ONT datasets. Here, we developed a novel pipeline, nanotei, that allows detection of TE-contained structural variants, including individual TE transpositions. We exploited this pipeline to identify TE insertion in the *Arabidopsis thaliana* genome. Using nanotei, we identified tens of TE copies, including ones for the well-characterized ONSEN retrotransposon family that were hidden in genome assembly gaps. The results demonstrate that some TEs are inaccessible for analysis with the current *A. thaliana* (TAIR10.1) genome assembly. We further explored the mobilome of the *ddm1* mutant with elevated TE activity. Nanotei captured all TEs previously known to be active in *ddm1* and also identified transposition of non-autonomous TEs. Of them, one non-autonomous TE derived from (AT5TE33540) belongs to TR-GAG retrotransposons with a single open reading frame (ORF) encoding the GAG protein. These results provide the first direct evidence that TR-GAGs and other non-autonomous LTR retrotransposons can transpose in the plant genome, albeit in the absence of most of the encoded proteins. In summary, nanotei is a useful tool to detect active TEs and their insertions in plant genomes using low-coverage data from Nanopore genome sequencing.

## 1. Introduction

Transposable elements (TEs) are a major component of plant genomes, and up to 90% of the genome can be occupied by different TE families [1]. Although the general impact of TEs on genome functionality is negative, they represent an important force of plant evolution, creating enormous genome variability [2]. The latest reports based on pangenome sequencing demonstrated that multiple traits involved in plant adaptation were tuned by TE insertions (TEIs) [3,4,5,6]. In addition, TEs make a significant contribution to the phenotypic diversification of crop species by creating new alleles, changing the gene transcription repertoire and triggering structural variations (SVs) [2,7,8,9,10,11,12]. Moreover, the inclusion of TEIs in association studies may bring new candidate loci associated with phenotypic variations, as demonstrated in tomato [9], rice [13] and other plants [11]. In tomato, for example, TEIs were associated with variation in major agronomic traits [9,12]. Taking into account the importance of TE transposition for plant genome evolution, local adaptation and domestication, the ability to trace the newly occurring insertions is crucial. However, the links between individual TE insertions and changes in different levels of cell organizations are poorly understood. The main challenge in the establishment of such connections is the technical difficulties of TE insertion detection and annotations. Most of the studies in plants have exploited short-reads to find TE insertions [3,5,9,10,14,15,16]. Next-generation short-read sequencing (NGS) significantly accelerated the discovery of active TEs and their insertions. NGS and accompanying bioinformatics tools allowed TEI detection to be performed in a high-throughput manner [10]. However, this approach is prone to miss multiple TE insertions [15], e.g., the short length of NGS reads makes TEI identification in repetitive and low complexity regions challenging. Furthermore, TEI identification based on short-reads has often used clipped reads to map the TE-genome junctions that further reduced the read length and resulted in poor genome mapability. Therefore, NGS detection of TEIs resulted in underestimation of TEIs and active TEs. Long-read sequencing technology, including Oxford Nanopore Technology (ONT) and PacBio sequencing, is a ‘game-changer’ for mobilome and repeatome investigation [17,18]. Compared to short-read data, long-read sequencing allowed the identification of a significantly higher number of TEIs in both heterochromatic and euchromatic [19,20]. ONT sequencing can be performed in a conventional laboratory with minimum investment in equipment facilitating experiment design and data generation. Even though the error rate of ONT reads is quite high, they have high mapability and may span an entire TE insertion [21]. Another advantage of ONT sequencing is that raw data can be used for epigenetic profiling of the genome [22]. Therefore, ONT-based TEI detection is more sensitive and accurate and requires smaller genome coverage [17,21,23]. Several tools have been developed for TEI identification in non-plant genomes, including xTEA [24], TLDR [25] and PALMER [26]. These tools were designed to detect insertions of various human TEs, such as L1, Alu, SVA and HERV, using data from different sequencing platforms. The application of these tools to detect TEIs in plants is not straightforward and needs additional bioinformatics expertise to adopt the default setting to plants. In addition, TLDR requires at least one spanning read per insertion, which requires a higher N50 value. PALMER takes PacBio data, and it was not tested for ONT reads. Thus, no tools for easy and automatic detection of TEIs and the corresponding original TEs using low-coverage ONT data have been proposed for plants.

To fill in this gap, we developed a new pipeline called nanotei (https://github.com/Kirovez/nanotei (accessed on 3 November 2021)) that performed reference-guided identification of TEIs from low-coverage nanopore data. The application of nanotei allowed detection of known and novel active TEs in the *ddm1* mutant of *A. thaliana* and unraveled tens of TEs missed from TAIR10 genome assembly. Thus, nanotei is a robust method for rapid detection of TEIs and the associated structural variants using low-coverage ONT data.

## 2. Results

### 2.1. Nanotei—A New Pipeline for Genome-Wide Transposon Insertion Detection from Nanopore Data

**Figure 1:**
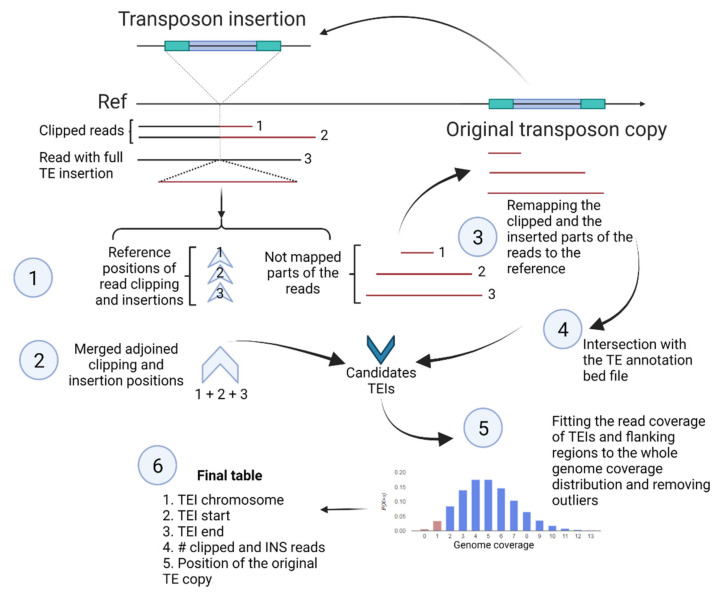
Schematic view of nanotei pipeline. The main steps are enumerated. The red parts of the reads corresponding to TE-contained sequences. Created with BioRender.com (accessed on 3 November 2021).

Although several algorithms have been proposed for transposable element insertion (TEI) detection using short-read data, no ready-to-use solutions have been described for TEI detection using the low-coverage ONT reads. Here, we developed a new pipeline called nanotei for the detection of TEIs and associated structural variants using low genome coverage Nanopore data. Nanotei requires four input files: bam file with ONT reads aligned to the reference genome, genome fasta file, fastq file of ONT reads and 4-column bed file with TEs annotated in the reference genome. The principle of this pipeline is illustrated in Figure 1.

First, the bam file is parsed to detect reads with clipped ends (default, >1000 bp clipping size) and full-length in-read insertions and to determine the corresponding positions on the reference genome. Second, the adjoined reference positions of the clipping and in-read insertions are merged if the distance between them is small (default, <20 bp). Third, the read sequences of the clipped ends and in-read insertions are extracted from raw reads and mapped back to the genome. Fourth, the mapping positions are intersected with TE annotations, and the best TE matches are collected. Finally, genome coverage by ONT reads is calculated, and then the number of reads supporting TEIs (clipped reads + reads with in-read full-length insertions) and the total number of reads in the TEI flanking positions are tested to fit the genome coverage distribution, followed by outlier filtering. The final table of nanotei contains the information about TEI position, the number of the corresponding clipped reads and reads with insertions, the id, and the genomic coordinates of the original TE copy.

Thus, we developed a pipeline for genome-wide detection of TE-contained structural variants (SVs), including transposon insertions using Nanopore data and information on TE annotation in the genome.

### 2.2. Numerous TE Copies Are Hidden in TAIR10 Genome Assembly Gaps

**Figure 2:**
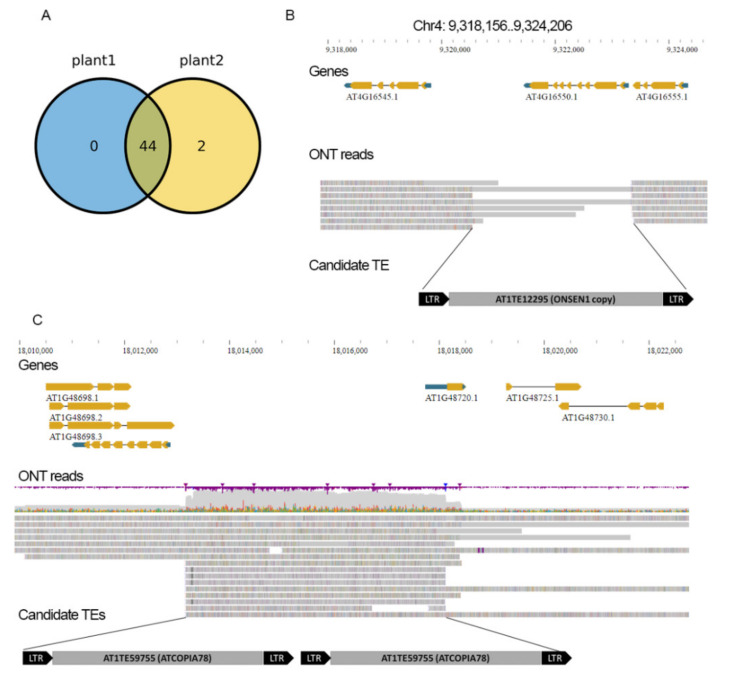
(**A**) Venn diagram showing the number of TEIs common between two Col-0 plants. (**B**) TEI on chromosome 4 and the schematic representation of the TE candidate AT1TE12295 proved by local assembly. (**C**) Tandemly organized TEIs with two ATCOPIA78 TEs, one of which was missed in TAIR10.

As a baseline to test nanotei, we used Nanopore reads of wild-type (Col-0) *A. thaliana*. We performed sequencing of two Col-0 samples, collected ~60,000 Nanopore reads (~7× genome coverage, N50 ~12 Kb) and ran nanotei. Unexpectedly, we detected 46 TEIs (colTEIs) from 43 distinct TEs (Supplementary Table S1). Most of the colTEIs (44) were common between two Col-0 plants (Figure 2A). To verify that found colTEIs were not specific for our *A. thaliana* plants, we used ONT reads from the publicly available dataset (NCBI accession: ERR5530736) as an additional control. We observed that all colTEIs are also detectable in this dataset. As *A. thaliana* genome assembly (TAIR10) includes at least 100 annotated gaps, we thought that the colTEIs could overlap with these gaps, being a direct result of incorrect genome assembly. In addition, such cases need to be filtered out because they will challenge the detection of real TEIs in our further analysis. To detect such gap-associated colTEIs, we compared the location of colTEIs with TAIR10 genome gaps (sequences with 3 or more ‘N’s) and found only 10 TEIs (22%) overlapping 10 genome gaps (Figure 2B). These results suggest that >40 *A. thaliana* TEIs are located in the annotated (10 TEIs) and unannotated (36 TEIs) genome assembly gaps. We further analyzed the classification of the TEs producing the colTEIs and found that both DNA transposons and retrotransposons contributed to TEIs. Although most of the subfamilies contributed to a single TEI, TEs from eight subfamilies generated 2–3 TEIs (Supplementary File S1: Figure S1). One of these subfamilies is ATCOPIA78, possessing the well-known ONSEN transposons that are involved in two TEIs located on chromosomes 1 and 4. We performed local assembly of the ONSEN TEI on chr4 (Chr4: 9,318,156..9,326,206) using ONT reads and compared the assembled contigs with the ONSEN1 transposon (AT1TE12295), which was assigned to this TEI by nanotei, as well as the border sequences of the TEI. The results showed 99% similarity of ONSEN1 to the assembled contig, pointing out that this ONSEN copy was missed in the TAIR10 assembly. Another member of the ATCOPIA78 subfamily with the newly identified TEI is AT1TE59755. We found that this element has two tandemly organized copies on chromosome 1, but one of these copies was missed in the TAIR10 assembly (Figure 2C).

Thus, the results of TEI identification by nanotei using Col-0 ONT reads provide evidence that tens of distinct TEs are missed from the TAIR10 genome sequence because of gaps and errors in the genome assembly.

### 2.3. Known and New Active TEs of the ddm1 Mutant

**Figure 3:**
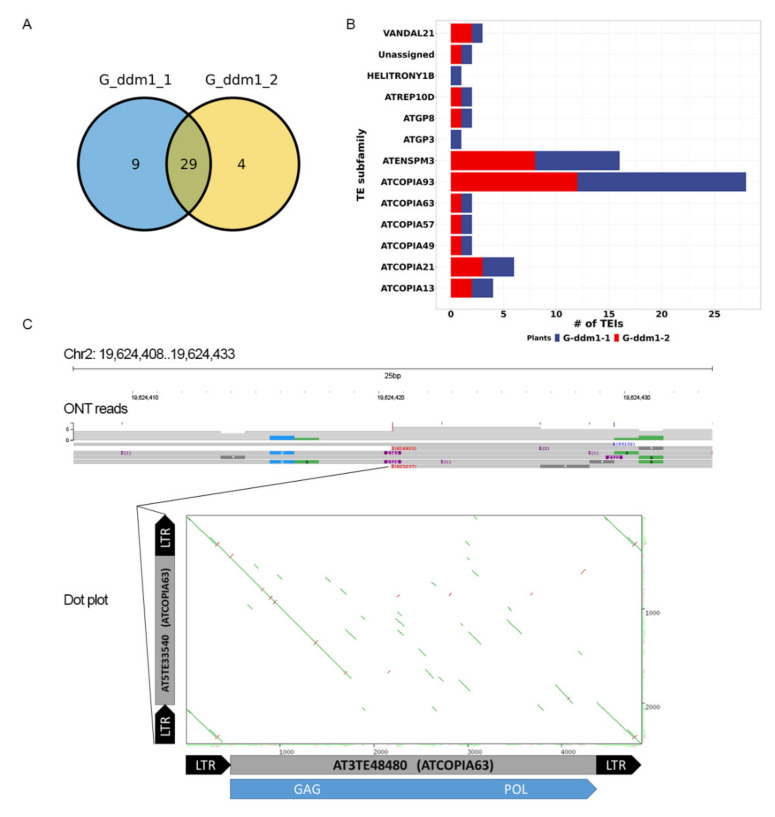
(**A**) Venn diagram showing the number of TEIs common between two *ddm1* plants. (**B**) Number of TEIs in *ddm1* generated by different TE subfamilies. (**C**) Read alignment and TEI site of AT5TE33540 and dot plot showing the sequence similarity with full-length TE AT3TE48480. The blue box in the dot plot shows the ORF encoding GAG and POL polyproteins.

We were interested in exploring the mobilome of TEs with ongoing activity. For this, we used *ddm1 A. thaliana* plants carrying mutations in the *DDM1* (decreased DNA methylation) gene, causing hypomethylation of cytosine in all contexts in *A. thaliana* [27,28,29,30]. As a result, some TEs are actively proliferating in *ddm1* plants [31,32]. We performed whole-genome Nanopore sequencing of two siblings of the *ddm1* mutant that have T-DNA insertions of the GAG fragment of the EVD retrotransposon (G-*ddm1*-1 and G-*ddm1*-2). We collected 74,792 and 80,260 high-quality reads with N50 ~12Kb corresponding to ~7× genome coverage evaluated after mapping of the reads to the TAIR10 genome. We ran nanotei with these reads and the TE annotation file [33]. After removing colTEIs, 38 and 33 TEIs were detected in G-*ddm1*-1 and G-*ddm1*-2 plants, respectively (Supplementary Table S2). Of them, 29 TEIs were common between the two plants (Figure 3A). Next, we analyzed which TE families are contributing to TEIs. Classification analysis showed that 15 TEs generated the *ddm1* TEIs belonging to 13 subfamilies (Figure 3B). Of them, 4 TEs (AT1TE42210, AT2TE20205, AT4TE18510 and AT5TE20395) from two subfamilies (ATENSPM3 and ATCOPI93) generated 63% (27) TEIs. The most active TEs in *ddm1* were EVD retrotransposon (AT5TE20395, 17 TEIs) and CACTA1 DNA transposon (AT2TE20205, 8 TEIs). EVD and CACTA1, as well as three other TEs (AT2TE42810 (subfamily VANDAL21), AT2TE23855 (subfamily ATCOPIA13), AT5TE65370 (subfamily ATCOPIA21) and AT1TE45315 (subfamily ATGP3)) were also previously shown to be active in *ddm1* by tilling array, Southern blot and short-read sequencing approaches [3,31,32]. We also identified non-autonomous TE AT5TE33540 from the ATCOPIA63 subfamily that produced TEI on Chr2: 19,624,409..19,624,434 (Figure 3C). The insertion from this element has been previously detected in epiRIL plants [34]. We found that this element possesses two LTRs and encodes a single ORF for the 562aa GAG protein, suggesting that AT5TE33540 belongs to the previously characterized Terminal-repeat Retrotransposons with the GAG domain (TR-GAG [35]). The similarity search between AT5TE33540 and other ATCOPIA63 members revealed high similarity to potentially autonomous element AT3TE48480 with a long ORF encoding a full set of TE proteins required for transposition (Figure 3C). Therefore, this element may provide proteins required for the transposition of AT5TE33540.

Using nanotei, we also found TEIs involving TEs that were not shown to be active in *ddm1* before. Manual curation of these TEIs showed that most of these TEIs are large structural rearrangements rather than TE insertions per se. However, we found one TEI at Chr4 (3,464,984..3,465,035) detected in both *ddm1* plants and fully covered by ONT reads (Supplementary File S1: Figure S2). Using the ONT reads from both *ddm1* plants, we performed local assembly of this region and obtained a 13,887 bp contig (Supplementary File S2). This TEI resulted from the transposition of a ~2 Kb length TE (AT2TE84980) from ATCOPIA57 (Figure S2). This non-autonomous TE contains two LTRs and no intact ORFs. The absence of any protein-coding capacity of this element suggested that its transposition may occur via the activity of proteins of other elements from the same family. However, ATCOPIA57 contains 48 elements in the TAIR10 genome [33] assembly with the longest TE of 2694 bp length and no autonomous TEs. Based on this, the mechanism of AT2TE84980 transposition is not clear.

Taken together, our mobilome analysis using nanotei and ONT reads from the *ddm1* genome allowed simultaneous detection of all TEs active in *ddm1* and revealed the transposition of two non-autonomous retrotransposons with one of them encoding the full-length GAG protein. Here, we provide the first direct evidence that TR-GAG elements are capable of transposition in plants.

## 3. Discussion

Many TEs are expressed in plants and have ongoing transposition activity, playing a major role in genome evolution, adaptation and plant breeding [2,18,31,36,37,38]. Detection of new TEIs is essential for a deeper understanding of TE biology and their multisided impact on genome architecture and plant diversity. Long-read data is a great tool to discover new TEIs and associated structural variants in plants [17,39,40]. However, no straightforward tools have been developed to gather this information from low-coverage ONT datasets. Here, we present nanotei, a pipeline that allows the detection of TE insertions and TE-contained structural variants using a reference-guided approach. To show the robustness of nanotei, we generated ONT genomic data for the Col-0 wild type and *ddm1* mutant of *A. thaliana*. Surprisingly, using nanotei, we identified tens of TE copies hidden in genome assembly gaps. Furthermore, one of the TEIs belonged to the ONSEN family, which has been investigated for a long time [41,42,43,44]. Eight copies of ONSEN have been previously described [41], with four copies making 90% of all insertions after activation by heat stress [45]. Our analysis revealed that an additional copy of ONSEN1, one of the most active ONSEN copies, is present in a genome assembly gap located on chromosome 4. Whether this newly identified copy has transposition activity and how it contributes to the heat-activated mobilome remains to be investigated in the future. Our results also indicate that current *A. thaliana* genome assembly requires revision, and new emerging technologies (ONT and HiFi long-reads, optical mapping, Hi-C scaffolding, etc.) may successfully assist this process [23,46,47].

We also traced the ongoing TE transpositions using the *ddm1* mutant as a model. Previous analysis [31,34] of the *ddm1* mobilome using tilling array, short-read sequencing and Southern-blot approaches found four TEs (AT2TE42810 (*VANDAL21*), At2g13940 (ATCOPIA13), At5g44925 (ATCOPIA21) and At5g17125 (EVD, ATCOPIA93)) from distinct subfamilies having higher copy numbers in *ddm1* compared to wt plants. Additionally, three TEs (At1g35370 (ATGP3), At2g12210 (CACTA1, ATENSPM3), At4g08680/At1g78095 (AtMu1)) exhibited higher copy numbers in *ddm1* based only on the tilling array. We checked whether the transposition activity of these TEs was detected in our *ddm1* plants and found that all these TEs were indeed captured by nanotei, implying that nanotei is a robust method to detect TE insertions and associated structural variants from ONT data. Taking into account that nanotei can identify TEIs from low-genome coverage ONT data (~7× in our analysis), TEIs of few *A. thaliana* plants can be easily captured by even a single MinION flow cell. For example, here, using barcodes, we sequenced 4 plants in parallel on a single flow cell and generated enough data for TEI detection. Therefore, ONT-based TEI detection captured with nanotei allows rapid mobilome characterization with a short turnaround time. It is worth noting that most of the TEIs identified by nanotei in this study were located in pericentromeric regions (Supplementary File S1: Figure S3) of *A. thaliana* chromosomes. These regions are enriched by different classes of TEs and other repeats that can hamper TEI locations by short-read data. A high mappability of long reads to the genome reference allows the identification of TEIs even in repeat-rich regions. We believe that nanotei will further facilitate the progress of comprehensive evaluation of TE activity and its contribution to plant genome, transcriptome and phenotypic diversity and evolution.

An important advantage of the approach described in this study is the ability to reconstruct sequences of full-length copies. This allows the identification of donor TEs. Moreover, with sufficient sequencing depth, the sequences of reconstructed TEs from insertion sites can provide valuable information for the study of the molecular evolution of TEs. This may include the diversity of protein coding capacity of new TE copies, accumulation of single-nucleotide mutations and structural variants, and distribution of methylation of individual TE copies [25]. For example, an intriguing finding of our investigation is the insertion of two non-autonomous LTR retrotransposons in the *ddm1* genome. Namely, we detected insertions of AT2TE84980 from ATCOPIA57 (Figure S2) and AT5TE33540 from the ATCOPIA63 subfamily (Figure 3C). This finding suggests that non-autonomous LTR retrotransposons can be a parasite of other LTR TE members to perform their transposition, such as BARE1/BARE2 elements [48]. However, AT2TE84980 belongs to the family with no members carrying ORFs for all TE proteins. Therefore, the transposition of this TE is probably assisted by TEs from another family. This implies low specificity of TE proteins to their original copies, but this is a poorly understood topic for retrotransposons. It is worth noting that these TEs are different in their protein-coding capacity. While AT2TE84980 has no long ORFs, AT5TE33540 has the entire ORF for GAG protein translation. This suggests that this TE belongs to TR-GAG retrotransposons with a single ORF encoding GAG. TR-GAGs were found in many plant species [35]. Our recent transcriptome survey in sunflower [36] and triticale [18] using ONT RNA sequencing found that these elements are transcribed. However, whether these elements are transpositionally active or only serve as a source of GAG proteins for other TEs have not been known so far. The detection of new insertions in the *ddm1* genome by our current analysis provides the first evidence that TR-GAGs can transpose, albeit in the absence of most TE proteins. This also highlights that TE evolution should be investigated as a network of functionally connected autonomous and non-autonomous elements [48].

## 4. Materials and Methods

### 4.1. Plant Material and Growth Conditions

Seeds of *ddm1* mutants (*ddm1-2*, F7 generation) were kindly provided by Vincent Colot (Institut de Biologie de l’Ecole Normale Supérieure (IBENS), Paris, France). *Arabidopsis thaliana* Col-0 plants (wild type and *ddm1* mutants) were grown in a light chamber for a month under 22 °C and long-day conditions (16h light/8h dark).

### 4.2. HMW DNA Isolation and Size Selection

High molecular weight DNA was isolated from 200–500 mg of fresh and young leaves that were homogenized in liquid nitrogen. DNA isolation was carried out according to the previously published protocol (https://www.protocols.io/view/plant-dna-extraction-and-preparation-for-ont-seque-bcvyiw7w (accessed on 3 November 2021)).

### 4.3. Nanopore Sequencing and Basecalling

Library preparation was carried out from 1 μg of DNA using the Native Barcoding Expansion 1–12 (Oxford Nanopore Technologies (Oxford, UK), catalog no. EXP-NBD104) and the Ligation Sequencing Kit SQK-LSK109 (Oxford Nanopore Technologies). Sequencing was performed by MinION equipped with a R10.3 flow cell. The sequencing process was operated by MinKNOW software (v.19.12.5). Basecalling was performed by Guppy (Version 3.2.10). Read mapping was carried out by minimap2 [49] to TAIR10.1 (https://www.ncbi.nlm.nih.gov/assembly/GCF_000001735.4/ (accessed on 3 November 2021)) genome assembly.

### 4.4. Nanotei Pipeline

Nanotei is written in python3 and can be run in Linux systems. The principle of nanotei is illustrated in Figure 1. In the first step, a bam file with mapped ONT reads is parsed, and the mapping positions of the following categories are extracted using the pysam package (https://github.com/pysam-developers/pysam (accessed on 3 November 2021)): reads with clipped starts and ends (S in CIGAR string) and reads with a detected insertion (I in CIGAR string). The unmapped sequences of clipped parts and insertions of the reads are extracted with the assistance of the biopython package [50] and mapped to the genome using minimap2 [49]. Then, the mapping positions are intersected with the bed file of TE annotation using bedtools intersect [51], followed by results aggregation using pandas package. Next, the initial bam file is used to estimate genome coverage by ONT reads using random sampling of genomic intervals and their coverage estimation by pysam. The obtained distribution is used to filter out TEIs with coverage that is too low and TEIs from regions with coverage that is too high. After this step, the final table with TEI coordinates and associated TEs is obtained. We tested this pipeline on a local server equipped with 500Gb RAM and 128 CPU cores. On this server, nanotei takes from 2 (low-coverage ONT data generated in this work) to 10 (ERR5530736 reads, ~40× TAIR10 genome coverage) minutes for the analysis of one sample.

### 4.5. Manual Curation of TEIs

To prove the presence of TEIs and corresponding TE, we collected the reads from the TEI region using the pysam.fetch() function from the pysam package. The reads were assembled by Flye assembler with the following settings: --genome-size 100K --threads 100 -m 1000. The assembled contigs were then blasted versus the TE candidate and TEI borders. When the assembly was not possible, the distinct raw reads from TEI sites were blasted vs the TE candidate. BLAST search was performed using Sequenceserver [52].

### 4.6. Statistics and Data Visualization

Statistical analysis was carried out in Rstudio Version 1.2.1335 (http://www.rstudio.com/ (accessed on 3 November 2021)) with R version 3.6.0. Visualization was carried out by ggplot2 [53] and ggvenn (https://github.com/yanlinlin82/ggvenn (accessed on 3 November 2021)) R packages. Read alignment visualization was performed in jbrowse2 [54].

## Data Availability

Oxford Nanopore reads generated in this study are available at NCB, project accession number PRJNA736208.

## Supplementary Materials

The following are available online at https://www.mdpi.com/article/10.3390/plants10122681/s1, Figure S1: Number of colTEIs per TE subfamily; Figure S2: Read alignment and TEI site of AT2TE84980 and dot plot showing sequence similarity with full-length TE; Figure S3: Circos plot showing the distribution of all ddm1 TEIs, annotated TEs and genes of *A. thaliana*; Supplementary File S2: Sequence of contigs assembled from ONT reads aligned to the TEI region on Chr 4 (3,464,984..3,465,035); Table S1: TE insertions identified by nanotei in Col-0 assembly using low-coverage Nanopore reads of two plants; Table S2: TE insertions identified by nanotei in ddm1 using low-coverage Nanopore reads of two ddm1 plants.
