# Supplementary material for: Transposons Hidden in Arabidopsis thaliana Genome Assembly Gaps and Mobilization of Non-Autonomous LTR Retrotransposons Unravelled by Nanotei Pipeline
Source: Plants (Basel). 2021 Dec 6;10(12):2681. doi: 10.3390/plants10122681 (PMC8704663; doi:10.3390/plants10122681)
Supplement: Supplementary file 1 [file plants-10-02681-s001.zip › plants-1470672-supplementary/Supplementary file S1.pdf]

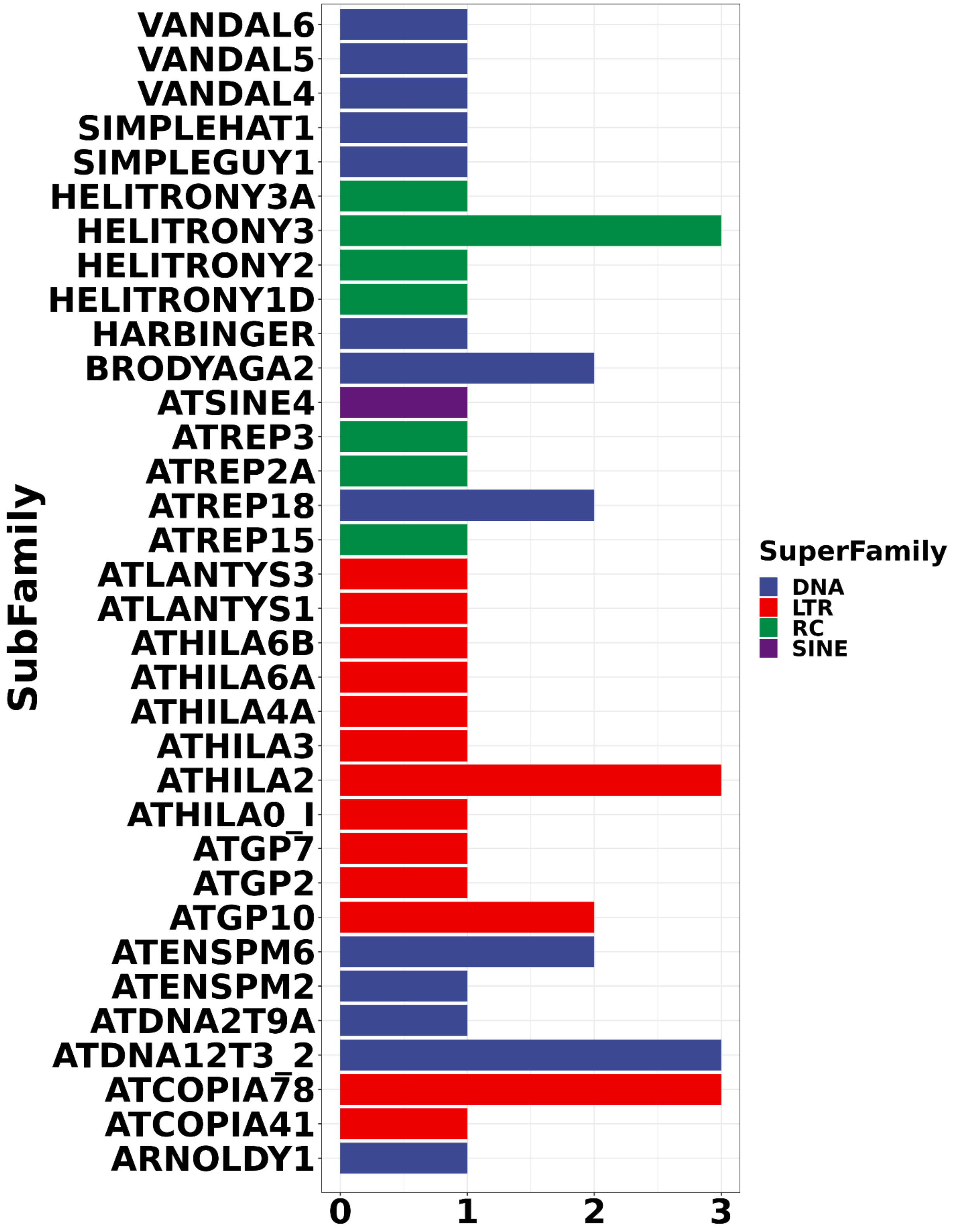

Supplementary Figure S1. Number of colTEIs per TE subfamily. Colors correspond to superfamily classification.

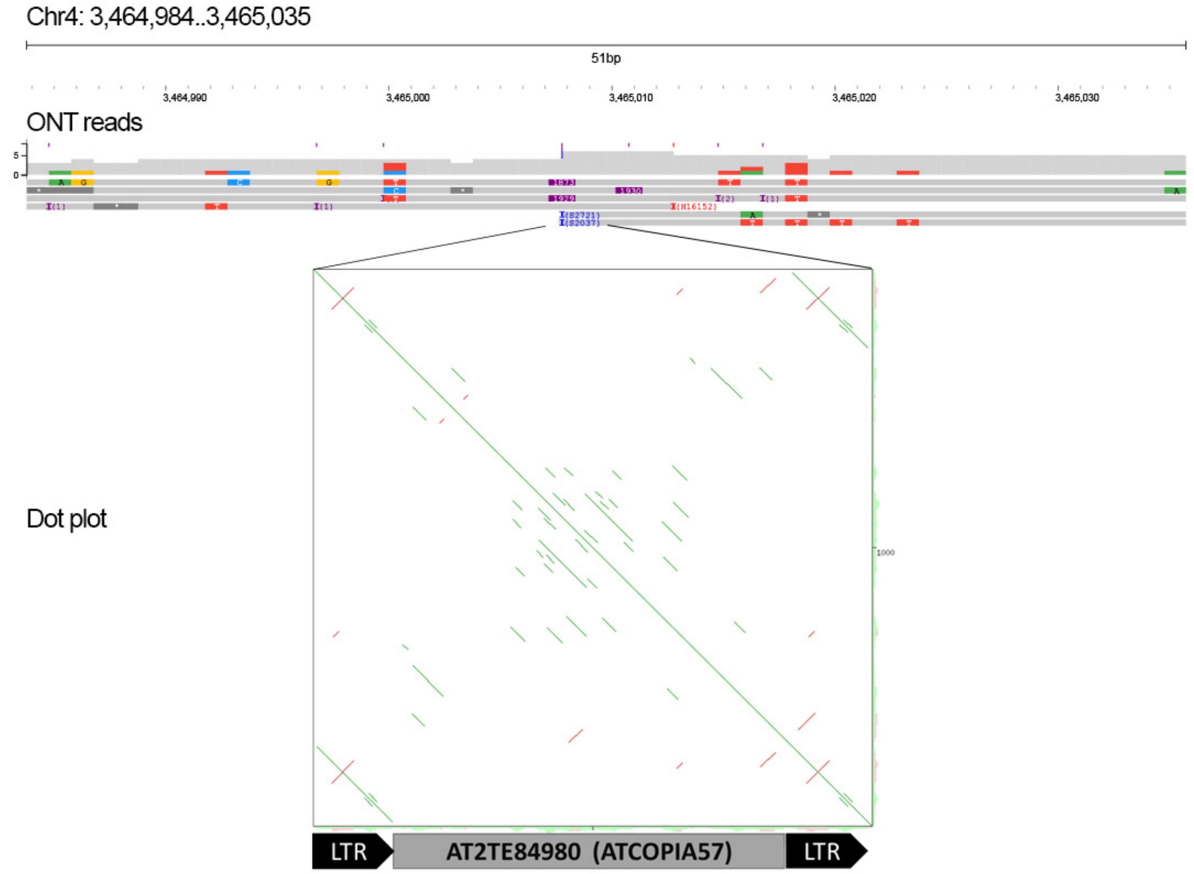

Supplementary Figure S2. Read alignment and TEI site of AT2TE84980 and dot plot showing the sequence similarity with full-length TE.

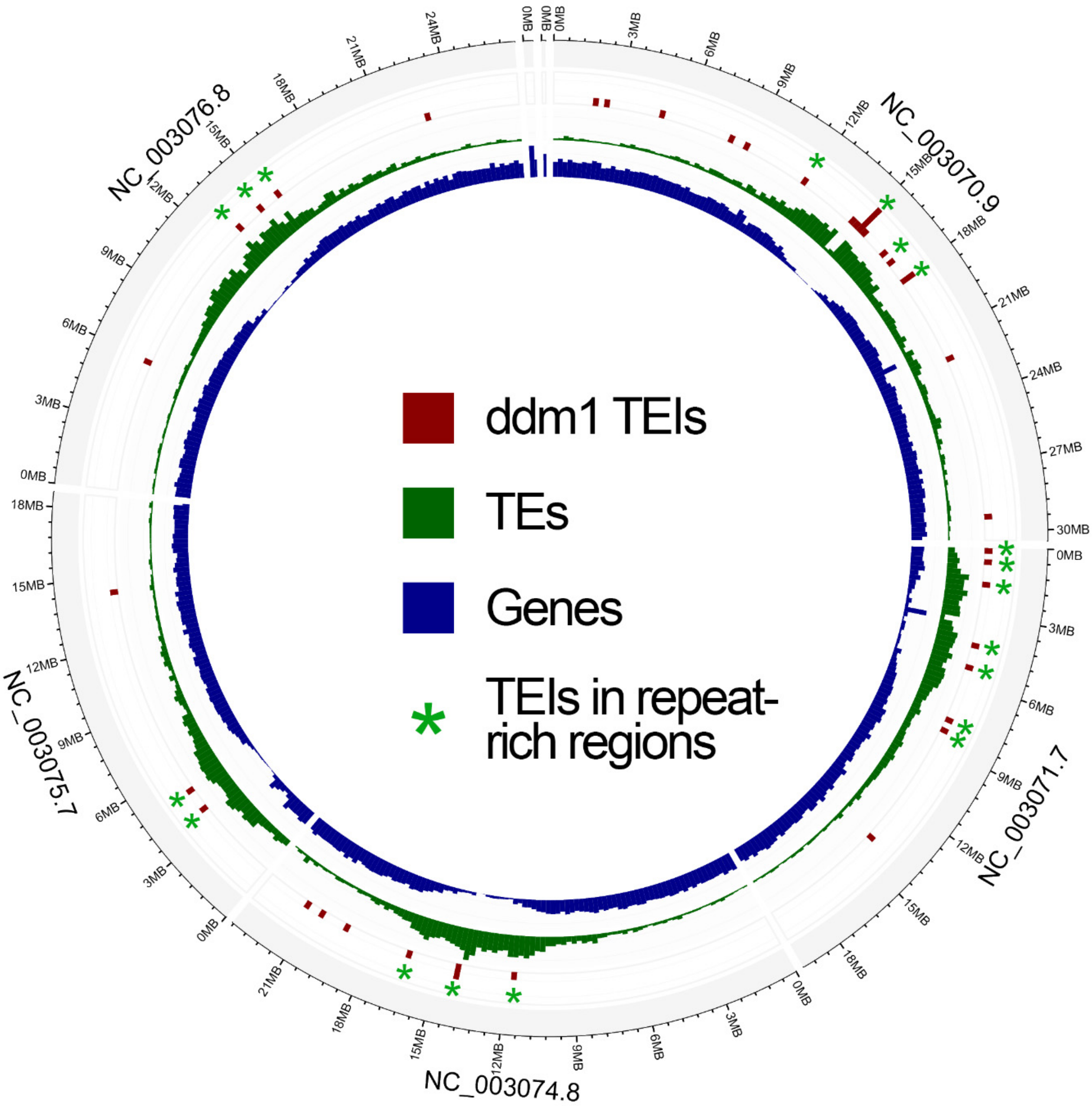

Supplementary Figure S3. Circos plot showing the distribution of all ddm1 TEIs, annotated TEs and genes of *A.thaliana*. TEIs located in repeat-rich regions (pericentromeric regions) are labeled by green asterisk.
