# Supplementary material for: Transposons Hidden in Arabidopsis thaliana Genome Assembly Gaps and Mobilization of Non-Autonomous LTR Retrotransposons Unravelled by Nanotei Pipeline
Source: Plants (Basel). 2021 Dec 6;10(12):2681. doi: 10.3390/plants10122681 (PMC8704663; doi:10.3390/plants10122681)
Supplement: Supplementary file 1 [file plants-10-02681-s001.zip › plants-1470672-supplementary/Supplementary file S2.pdf]

Supplementary File S2: Sequence of contig assembled from ONT reads aligned to the TEI region on Chr 4 (3,464,984..3,465,035). The inserted TE element is colored by yellow:

>contig_chr4_TEI

CCTTGTTGATTGCACTTTTATTTACGCCTTATTTGCATCCCTATTTGTATCCCCTGTTTA

TTGCATTTAGGTTACTTAATTTTGCTGCCTGTTGGTTCTACTCGACCCAGTGATCGAGTA

GGGCTCTTAAGTTCTTATTTTTACTTGTTTGAATCCTGACCTAGGATTGTTACTAGAAAC

CCACCATCATTATAATATGGCTTGACTTAGTTGTTTTTGTACACATCTTGATTGCTAGCA

ATACAGTGGACTAGATTGACATCCTCTATAACTGCAACGACATGTTTGTGTTTTAGGAGA

ATTGAACTGAATCACACATTCACCATAAGCAGACCTTAAAAACCTGCTTATCAATTTGGC

GGCATTGCCAATTCATTGTTGCATTGTTACATTCAAGATATCAGAAACTTTTAAGATCAA

GTTCTTTTACATTTATCAAGTTACTAACTCATCATTTCGTCTGCTTGTTTTTGTGGTATA

GGTACTAATCTTTGTGCATGCAAACTCCATCCAAAGGTTCTGCACAATCAAGACAGTGGA

CTAGATTGACATCCTCTATAACTGCAACGACATGTTTGTGTTTTAGGGAATTGAACTGAA

TCACACATTCACCATAAGGAGACCTTAAAAACCTGCTTATCAATTTGGTGGCATTGCCAA

TTCATTGTTGCATTGTTACATTCAAGATATCAGAAACGTTTAAGATCAAGTTCTTTTACA

TTTATCAAGTTACTAACTCATCATTTCGTCTGCTTGTTTTTGTGGTATAGGTACTAATCT

TTGTGCATGCAAACTCCATCCAAAGGTTCTGCACACCTACTACCATTCAGAGACACAATT

GACAAAATAGCTCATGAGTTACAAGAAACCAAAGTAAAGGCAGCCTGTGATCAGCAAAGA

CCATCTGCTATGGATTAACAGAACAGACCAGTTGATGTTCAAGACCCACCTAATGTTGAT

CAACCAAGAAACATTGGTGCTGGTGATGCCCAAGGAATCATCACCAAAGACAAGGGATAG

TGCCACCACCAGTCCAGAACAACAACTTTGAGATCAACAGTGGTCTCATCTCCATGATCC

AAGGAAACAAGTTTCATGGTCTACCTATGGAAGACCACCTGGACCATCTTGACAGCTTTG

ATAGGCTTTGTGGCCTTACCAAGATCAATGGTGTCACTGAAGATATGTTTAAGCTCAGGT

TATTTCCCTTCTCTTTGGGAGACAAGGCACACCACTGGGAGAAAACTCTACCCCCAAACT

CCATCACCTCATAGGACGATTGCAAGAAAGCTTTTCTTGCCAAGTTTTTCTCTTAACGCT

CGCACCGCTAGATTGAGGAACGAGATCTCAGGTTTCAACCAGAAAAATAATGAAACTTTC

TGTGAAGCTTGGGAGAGGTTTAAAAGCTACACCACTCAGTGCCCCCATCACGGTTTTAAG

AAGGCTTCATTATTGAGTACACTCTACAGAGGAGCCTTACCAAAGATCAGAATGCTACTT

GACACCGCCTCCAATGGAAACTTCCTGAACAAAGATGTAGTAGAAGGCTGGGAGTTGGTT

GAAAATCTAGCACAATCTGATGGGTGCTACAAAAGACTATGATCCCTCAATCAGAAGAAC

TGGAGGAAGTGAGGACAAACAGAGCAAGGATATCAAGGCTCTGAATGAAAAGTTAGACAA

GCTGTTGCTGGTTAGCAGAAGCAGATAAATTACATCACTGATGAAGAGCACTTCCAAATG

CAAGAAGGGGGAATGATCAAACTGAAGAGCTGTGCTACATTCAGAACCAAGGAGGGTTTA

ACAAGGGCTACAACAACTACAAGCCCAACCCAAACCTCTCCTACAGAAGCACCAATGTAG

CTAACCCCAAGATCAAGTGTATCCACCACAACAGAATCAGTCCAAGCCTTTTGTTCAGTA

CAACCAAGGTTATGTCCCTAAACAACAGTTCAATGGTGGCTACCAACAGCAGAATCCACC

ACCAGGGTTCACTCAACAACCACAACAAGCCCTAGCAGCCCAAGATCCTGACACAAAACG
AATGCTTCAACAAATCATCCAGGGTCAAACTACTGGAGCTCTAGTTCTGGAAGAACGATT
GGCTGAGATTAATAGTAAAGTTGACTGTTCCTACAATGAGTTAAGAAGCAAATATGAGGA
TCTCACATCTAAAATGACATACATGGAACGCCAAGCTGTTGCCAATACCTCCTCAACGTA
TCCAGGGCAACACCCAGGAAAAGCCATTCTAAATTCCAAGGAATATGCACATGCAGCCAC
ACTCCGTAGTGGAAGGAAATTGATCAACAACCAGCCAACAGAAAAGATCACTTAGGACAG
TGAAGTTCAAGAAGGGAGGACCAGCATCAAAACGAAGTTCAAACCGATGAACCAATTAAG
CTTGATCAGACCTTCAGACTCACTCAACCTCTACTCGATCAAGCAAAGCCAACTTTTGAG
GAAAGGAAAGCTGCGGCTACAGAGAAAAATAAAGAATTTGCTCCGCCACCCTTCAAACCG
GCTATGCCTTTCCTAGGAAGATTCAAGAAGGAATTAATAGAAAAATACAAAACTCTTTTT
GATAAGCAACTAAAGTAGATTGAGCTAAGAATACCATTAATGAATGCTTTCACGCTCATT
CCACACTCCCACAAGTACCTCAAAGATCTGATTATGGAAAGAATTAAGGAGGTACAGGAA
ATGGTGGTAATAGGCCATGAATGCAACGCTATCATCTAGAAAAATATAATACCAAGAAAG
TTAGGAGATCCTGGATCCTTCACCCTACTTTGTTTAGTAGGACCATTATCTTTCAATAAT
TGCCTATGTGATTTGGGTGCCTCAATCAGCCTCATGCCCCTATCTGTAGCCAGAAAATTG
GGTTTTAATAAATACAAGCCCTGCGGTATCCAACTGATATTAGCTGACAGGTTAGTCAGA
ATACCTCATGGAGTTCTCGATGACCTGCCTGTTAAAGTCAGATCAATAGACATCCCTACT
GATTTCGTAATTCTGGAGATGGATGAGGAGCCAAAAGACCCATTGATCCTAGGAAGACCA
TTCCTAGCTACTATAGGAGCTATCATCGACGTCAAGAAGGGAAAAATCGACCTAAACCTG
GGGAAGTAGATGAAAATGACCTTCGACATTAACAAAGCTATGAAGAAACCGACAATCAAT
GGATAAGTCTTTTGGATCGAAGAAATGGATAGATTAGCTGACAAATTACTGGAAGAACTT
ACAGAAGGAGATCACCTAGCAAGTGCCTTAACCAACGATGGAGAAGAAGGGTACCTACAC
CTAGAAACCCAAGGGTACAAAGAGTATCTTGACGCCCACATACCAATGGAAGGACCAGAA
GTGTTTGAGGAATTGATCGTTCCCTCAGAAGAAACAGTCTCAGGATGCTTCATGTGCCTA
ATTGTTGAAAAAACAAACTCTACTGAGATGCTCGACCATGGAGGGGAGAATATAAGTTCA
TATGACTAGTCAGAACTCAAAGTTTTAAAGGTTGACCTTAAACCTCTTCCAAAAGGTTTG
AGGTACGCGTTTCTCAGTCCAAATGATACTTATCCCGTCATTATTAATGATGGACTGAGT
GTTGAACAAGTGAACCAGTTGTTGAATGAACTTAGAAAGTATAGGAGAGCAATTGCGTAT
TCTTTAGCTGATATTAAAGGAATTTCACCTAGTTTATGTACTCATAGGATCCATCTTGAA
AATGAATCATATACTAGTATTGAACCACAGAGGAGATTGAATCCAAATTTGAAAGATGTG
GTTAAAAGGAAATTCTTAAATCGCTTGATGCTGATATAATCTACCCAATTTCTGACAGTA
CATGGGTCTCTCCTGTGCATTGTGTTCCCAAGAAGGGAGGAATGACTATAGTTAAAAATG
ATTTAGATGAGCTCATTCCCACTAGAACTATAACAAGACATAGAATGTGCATTGATTACA
GGAAACTAAATGTTGCAACTAGAGAGGACCATTTTCCTTGCCATTCATTGATCAAATGTT
AGAAAGGCTAGCTAACCATGTTTATTACTGTTTTCTTGATGGCTATAGAGGTTTCTTTCA
AATTGCAATTCGCCCTAATGATAAAAAAAAACCACTTTCACCTGTCCCTATGGAACCTTT

GCTTATAAGAGAATGCCTTTTGGTTTATGTAATGCTCCTGGGACTTTCCAGAGAAGCATG
ACTTCGATATTCTCGGATTTTATTGAGGAGATAATGGAGGTATTCGTGGACGATTTTTCA
GTCTATAGATCCTCTTTCTCCTCATGTTTGTTAAACTTGTGCAGGGTTCTAGAAATGTGT
GAGGAAACCAATTTGGTACTCAACTGGGAGAAGTGTCACTTCATCGTTCAGGAAGGAATT
GTGCTAGGGCATAAGATTTCTGGTAAAGGAATTGAAGTTGACAAGGCAAAATCGATGTCA
TGATTCAGCTGCAACCCCTAAGACAGTTAAGGATATCAGGAGCTTCCTCGGCCATGCAGG
ATTTTACAGGAGGTTCATAAAAGATTTTTCAAAGACTGCTCGACCCCTAAATAGACTGCT
GTGCAAGGAAACTGAGTTCAACTTTGATGAAGATTGTCTCAAAGCTTTCCATTTGATAAA
GGAAGCATTGGTGTCCGCCCTATCGTTCGAGCACCCAACTGGGACCACCCATTTGAAATC
ATATGTGACGCATCCGATTATGCAGTTGGAGCTGTCCTAGGCCAAAGGATTGATGGCAAA
CTTCACGTCATCTACTACGCGAGTAGAACGATGGACGAAGTTCAAACAAGATATGCAACC
ACAGAAAAGGAGCTATTAGCTGTTGTTTTCGCCTTTGAGAAATTTAGAAGTTATTTGGTT
GGTTCCAAAGTGAAGGTCTACACAGACCATGCAACACTAAGGCACATCTATGCCAAGAAG
GAAACCAAGCCAAGACTTCTAAGGTGGATATTGTTGCTTCAAGAGTTTGACATGTAAATC
ATTGACAAGAAAGGTGTTGAGAATGGAGTTGCAGACCATCTCTCCAGGATGAGAATTGAA
GATTCGGTCCCGATAGACGACACTATGCCTGAAGAACAGCTTATGTTCTACGACCTTGTT
AACAAAAGCTTCGATTCAATGGACATGCTGGAAGAAGCATATGGAGTTGAAGAAGAAAAG
CTGCCCTGGTATGCAGATTTAGTCAATTATTTGGTAAGTGGTATGATCCCCTAGAGTTTG
GATGCATATAAAAAGAAGTTCTTTAGAGACATCCATCATTACTATTGGGATGAGCCGTAC
TTGTATAAAAAGGGGAGTGATGGGCCATTCAGGAGCTGTGTCTCTAAAGAGGAAGTTCAA
GGTATATTGGGACATTGTCATGGATCCACCTATGGAGGGCATTTCGCAACCTTCAAAACT
GCTTAGAAAATTTTGCAAGCAGGTCTGTGGTGGCCCACGATGTTTAAGGATATTCAAGAA
TTCATAGCGAAGTGCGATCCATGCCAAATAGTTGGGAACATATCCAGAAGGAATGAGATG
CCACAAAATCCAATTTTTGAGGTAGAAGTTTTTGATGTGTGGGGAATAGACTTCATGGGG
CCGTTCAACCCTTCCTCATATGGAAATGCTTATATCCTGGTAGCAGTGGATTACGTCTCC
AAGTGGGTGGAAGCAATAGCAACACCAACCAACGACCACAAAGTAGTCCTGAAAATGTTT
AAGTCCGTTATTTTCCCCAGGTATGGTATTCCGAAGGTGGTGATAAGTGATGGTGGCTAA
CATTTCATAAACAAAGTTTTTGAAGGTCTATTAAGAAACATGGGGTTAAGCATAAGGTTG
CCACTCCTATCACCCTCAGACCAGTGGCCAAGTAGAAGTGTCATACAGGCAGATCAAGGC
TATAGTCACAAAGGTTGTTGGTGTCTCAAGAAGGGATTGGTCAGCTAAACTTGATGAGAC
TTTATGGGCCTATAGAACCGCATACAAAACGCCTATTAGGAGAACCCCATTCGAGATGCT
ATATGGGAAATCTTGTCATCTACCGAGTTGAAGTGGAATATAAAGCCATTTGGGCAACCA
AACTTTTGAATTTGGAAATCAAGGGAGCTCAAGAGAAAAGAGCAGTCGACCTGCATGAAC
TAGAAGAGATTAGGCTGGAAGCATATGAGAGTTCAAACATCTACAAAGAAAGAACAAAAG
CTTTTCATGAAAAAAGATCTCTCCAAAAGATTTCAACATTGGAGACCAAGTTCTGCTTTT
GAACTCCAAACTCAAGTTGTTTCCAGGAAAGCTAAAATCCAGGTGGTCTAGGCCTTTCAC

CATCAAAGAGATACTGCCATATGGGACAATTACTCTTACTAAGAAGGGAGGTTATGAATT

TACTGTGAATGGGCAGAGAGTTAAGCGTTATATGGCAGATTGTCCTATTCCTGAAGGAAC

TACAGTTGACCTCCAGGAACCCATCAATGCCTAAAATAGTAAGAAGTCTAGCTTAAGACT

TTAAACTAGCTCACTTGGAGGAAATCCCATGCCTATATCTGTACAGATTTAATTTTGATA

TTTTGATGTGCTTTTTAGTGTTTTTGGAATTCAGGAAATAAATCAGGATTTATGAGCTGT

GAACTCTGTGATCTAGAAATATTTGCAACAGAAGCAATTCTACTCTATCAATTGGTCGAG

TAAAATTGCATCACCATTGTACTCTATCAATTGGTCGAGTAAAGCAACTAAACGACCAAG

CTTGATGATATAGTGGCCCACTTGTCTCCCCATCATTCTATTACTCGGTGGACAAAGCCT

TTACCCATCATGCTCAATCACTATTTGGTCAACACCCTTATCCCAACAAAGAAAGCATAG

GAGACCATCCTCCTCCACTCATTCAAATCGAAACCTAAAGATAAGTTATCAACTCTCTCT

CACTTTCTTCTTTGTGCGCACTTCAATACCATCTTTCTCATTTGTTTTCTGCTTCACACA

ATCTCGAAAATTTTAGTTTTCAGCTTCCTCTAAAGAAAAAATCACTTGCCCCCTATTTCA

CTCGATCAAAATCGCAAGTATTTTTCTTTGTTTGAGACTTATCTACTAAGGTTTAGTTTT

GATTTTTGAGAGTTTAGCTTCACCATGAGTTCAAACAGCAACGAATCCTTCATGAACGCC

GATTTCAACGTGGATGAGGCCGAATCTTGGTATACTAGGCCTGAGAGGGAGATGGAAGAG

TATAGGCGGTTCAGTCAACATGCCACCAAGATCCTAGCTCGTGATAGAAGAAGAGAGGAA

ATTGCAAGAGGTAAGAGAGCTATGGCCGAAGAGAGAAGTCTCGTCGATGAATACCTTGGG

GGATGAAGATTATGTCCCTGAAATAACTCCAAGACTCACTAAGTCCTTGATGAAGAAGAC

TAAGCTATCACCGGATGGATACTATGAGCTTTTGGCAGCTCACAATTTCCATGGGACTCG

ATACCCCACTCTGAAACCATGAATGAGCTTGGGATAACAGAGGATGTCGAGTACCTCTTT

GAAAAGAGTGGTCTTCTGGGTCTCATGTCCAACCCTCACTCAGCCTACAAAACAGAGGCG

CTTCAGTTTATTGCTTCGCTGGAAGTAAAGTTGTTTCAGGGGCTTTCTAGCCATGAAGCA

AGAGAAGAAGGGCTTGGGTATATCACATTCGAAGTCTATGGAAAAGACTATGTTTTGGCT

ATCAAAACTCTTGAGGATATGTTCGGATTCCCTAGAGGGACTGATGTTAAACCCAAGTTC

AAGAAAGAGGAACTTTCGGATCTTTGGGTAACAATAGGTGATGACGCTGTATTCTCATCC

TCATGGGCCAAGAGCAACGCAATCCAAACCCCTGCATTCGGTACTTCCAGAAAGCAATGG

CTAACGTCCTCTACGCAAGGGAGAAGACCGGACCGATTAACAATGGTGAGATAGAGCTGT

TGGATATTACCCTAAAAGATATCCTAGTCGACACCAAGAACAAAGTGCC

TATGAAGGGTG

ACACCTGTTGGTTTATTGAGAGAAAGAAGAAAAACAGAGTAAGAGAGACATATCTCTTCT

CTTGTGAAGAAGACAAGAACAAAACTTTGTTTTATTGAAACTGAAACTTGTTAATCTTTA

CATGACGATACAAGGTGTTTATATAGAAAACAAATAGACACAACAACTCAATTAGTTCTG

TCACTTCATACTTGTGTCTCTTTCTACTTGTGCCTCTTGAGTGAAACAACACAAACTTAC

AACCTTTAACTGAAAACTCAATTACCGACCAACACTCCCCTTAATTGAGTTTCGGTCTTG

CACTTAGCTTCCTTCTCAGTTTGTCACAAGTTGACCTTGTTTACTTCTTCTTCCCTTGCA

AGTAAATCATTTGTGTTTTCTCCATCTCAATGGAAAACTCTTATGCTTGATCTTAATATC

CTGTATACTTCTTCCTTGAGGATAATTGATGAGACATCTGACACTAGAAACACCTCTTTG

ATGATCTTCTCTCCTTGGCTTGTCATCACTTGGATATTTCCTTTTCCATGTCTCATCATT

CATGATGATGATATTTCTTCAACTTGAGGTTCTTCACCACGTGCCTTGGCTTGTTACACA

CAAAGCACCTTCTTGTTGCAACTCGTATTTGACGTTGCACACTTTTGAGATTTCTCTTTC

CCACAACGCTTCTCGTTTGTCACCTTCAAATTTTTTTTTTTTCACGTGAGCTTTGTCACG

TCATCTCCGGTTGTTCTCACAACCTTTTTTCCACTCACTTTCTTTTAAATGAGATTACTT

CTGATTTCCTTTTGGTGTCCTCTTGATCACCAAATCTGTGCGTGAACTCCTCCACGTCTT

CTTCGGTGTCTTCTTTTGATCACCATAACGTCTCCATGACGTCTTGTGCGTGAATCTTCT

TCACGTCGTCTTTGGTTTACTCTTCAACCGACTTATGCTCCATTTTTCTCCGAGGTAAGC

ATCCTCTTGTGCTCCATTTTTCTCCGAGGTAAGCATCCTCTTATGGTGTCCTCTTTTGAT

CACCACCACGTCTTTTCGACGTCTTGTGCGTGAAAATCTCTTCACGTCTTCGTCGGCTAC

CTTTGCAACCGAACTGAACTCTGCTCCCTCTCTCTTAGGTAAGCAGCTTCACACACGTAA

GCAAGCTAACATCCCTGTGTTGCTTTCTCCAACAATCACCTCAATGTGATCGTCACACGT

ACAAATGTCACACCATTTCTTGGCCTTCTTTTGACTTTTGTCGCCCCGTTTGTTTCTTCC

TTTTGAAGCAAACTTTTCTCCACGTTTTCGTGTTGTAGTCATCTTCACAACTCAACACAA

CGTTTTGATAATAAGCCTTGCTCTGTTTGTTACAACAGCGGCTTTGCCGTCTTGCGCTTC

ACCAAGACGTTGTTGAACAAATTTTCAAAACTCTCCTTCCACAACTCCAAATCCACATTC

GTTTTCTCCATTGCCTCTAGAGAAGATTTCCTATCTTCTTCATCGCTTTTGGAGCCTTCC

TCTTGAAGGTACTTCATGTGAATGTGTTTGTGAAAACTGATGGTACTCTCTGTAGTTACC

ACCTCTTATTTCTTCTTCTTCCCTCTTCTTCTTCCTCTTCATCTCACCATAGATTTGTTG

TCTCCATCTCTCTTTTTCTTTTCGCAGCAGAACACATCTTATTTTTGTAGAGACAACCAA

GGACAACCATCGGCTCTGATACCATGTTGGTTTATTGAGAGAAAGAAGAAAAACAGAGTA

AGAGAGACATATCTCTTCTCTTGTGAAGAAGACAAGAACAAAACTTTGTTTTATTGAAAC

TGAAACTTGTTAATCTTTACATGACGATACAAGGTGTTTATATAGAAAACAAATAGACAC

AACAACTCAATTAGTTCTGTCACTTCATACTTGTGTCTCTTTCTACTTGTGCCTCTTGAG

TGAAACAACACAAACTTACAACCTTTAACTGAAAACTCAATTACCGACCAACAACACCAA

CGACGCT

TCACCATCAATGCGTTTATTGAATCATCTTTGTGGTTTCAGGAAATGGGCACT

AGCAAATAAGCAGAAGAGAACCATTTCTATAGGAGGGGTGATAACACCTACACTAATGGC

TTGTGGAGTGCCACTTCAGTCGACTCCCTTTGCCCCTAGATGGTGAGATGGGCTTTGTTT

ATTGATCATCAGAGTCATGAGGGGATGCATATTCTCAAGTTTCAGCATAGAGCGGAAATG

GATGGGAGGTTTCTCCTACCCAACTAAGAGCTCACAACCATCACAGTGAGGGGTAACATC

GATTTCAACCCGCCTAATGAAGAACTCTTCTTCATGGAACGAGCACCCCGACGAGAGAAG

CTCCAGACATTGAAGAAAGGGTTGAGAGTGAAGGAGAAGCTGAGAATGAAGAAGCAGAGG
AGATGGATTGGGAGAACTACAATGCAAGTATGTTCCATTTTGAGGAACACAAACCACCAC
CAAGAGTGAGTAAGAGTCTGACTGTTGCACATAAGAATATTGGTTTGATGAGTACTTGAA
ATAAGTTTCAAGACAGGATGTTGGAAAAATGTGGCAAGGCCATAGCCGCCATTCAGGCTG
TACTAAGCTTTTCATCTTCAGGCGCCACCATGGTGAGAGAAAACCGCCCTGGAGAAGTGG
TTTCAAGAAGGCATAGAGTTTCACCTTCAAGACAATCTGCCTATGAGAAACGAGAGGTAA
GTCGCCCTCAAGCTCCTGCACGACATTCGTCTCATGAACACCGCGAGCAGAAAAGAAGGA
GAAAAACAAGAATTGTTCGGCCAAGGAGCAAGGACCTTCTTATGTTCGGCAGAAGATCAC
TCGACCAAGATACTCGACGCGATATCGAGCAGAGTGTCGAGCAGGATCCGTGGGTTCAGA
ACGAGCAGAACGTCGAGCAAAATCAGTGGGATCAAGATGGGCAGTACTCAGGAATGAATT
GGGATGCTTACTATGGCATCAACGAGCAGGAACCTTCACCTGAAAACTGAGGTAACACCA
CCTCTCTATTGTATATACCATAGCATCTTTTTATTTTTCTTTGTGTGTATTGTGCTTTGG
TCTTGGATTCTCTTTCAGATTTTTATTACACAAGAGATTGTGTAATTTAAGTTTGGGGAG
GGTTCAAGACATGTTTAACTTTGTGTTCTCTTTTCTTATTTCAAAATTTGCATCATCTAA
GGCATAGAAAACAATAAAAAATTTGAAAATTTTTCAGAAAATTATTTCACAAAAAGAGTG
TCATCTAGATTGCATATCATTTTAGGATTGTATATAGAGTGTTAGCATTTAGGATTGCTC
ATTTGCATAGGGGATAATGATGATTTGAAACCGATAAGCATGTTTTGATTCAATAAAGTA
AGTTCAATGCCCAGGTTGTTAGTTCTCTGTTGTATAACCGATTGTATCTCTGAAATAAGA
CTGCACCCTGCTTTGAATTTGTAACTTGATTATACTACTTTGATCGAAACTTGCTTATTT
GATGCCATTCCTATCTATTAGAACCTGAACTGAATTTGAAATTATCATGTCTATGCTTTG
TTTATGAACTTATGGCTACCATATACATACTTGGATCATCTTATTCACTTCATCACCCTC
TGTTAATCCAAGTAGTTGTCTCTCATTTTTAGAGCAGTTTTCCGTACCCTTAGTCTAGCC
TTATTTCAAGGCAAAGATCATTTGTGTGTGATTGTGAGGTCTTTTTGGATTGAGCTTGGT
ATAACGTGTTAGGTATGAGCCGACAAGAGCAGTTTTTCATGTATACCTAGCCCACACTTT
TTGGGTTACCTAGGACTACGTGGAGACTTGTATTTCGGATTGAGAATGTGAAAGGAATGA
ATGGGAATGAGAGTGAGAATGAAAAAAAAAAAAAAAAAAAGAGAGTAAGAGTTTAGAGTTC
TAAGGGGATACAAAAGTGTTAAGTCTAGTAAATGTTTTCGAATTCAAAGAAGTTAGACAT
AGTGATTCAAAGAAAAGGGTAAAACGCCCTATGTGCTAAGATTAGAAGAAAAGCCTTAAG
TTTGTCAAAGTATAAAACCAAACCCGCTAGACTCTTAAATTGTTTCAAGAGAACCCTGTA
GAACATAAACCAAAAAGAGAGAAAGAATGATAAAAAGGGTAGAGTTAGGACATCTATGGG
AATGGGATCAGCTTCTGAGCTATCATACTGTATCATTGGGTAGATGGGAGTCTGTTCTTG
TATGTGTCTGTTGGCTTTTACCTTTAGCATTCTACTGAAGCTCAATCTTTTCTCTAAGGG
TCCCTGTTACTCGACCAAACCATTAGAGGGACCATTTTTGTCTCTTGGCCTAAGCCCAAA
ACCAAGTGAGTTCATAACCATTGCATTGTTTGATCCACTATCCGTGCTTAATGAATGTTA
AAGGGAATTGGTTGATGTGAATGCTTGAATAGATTAAGGCAAGTTGTTAGGTTGCATTGT
GACGAGTATGGCTAACATTTTTAAGTAAGGGTCTATCATCTTGCAACCTAGAATTAGCTA

CCTGGACATTGAGCTTACCCATTTTATAGGCATGCTTCGGTTTTTGAATCCCCACCTTCA

AACCTCTCCTTCAACTTAAGATTTTTTGTTTACTTGAGGGCAAGCAAGAGATAAGTTTGG

GCAAGCAAGAGATAGGTGTATGTTTATCATCATTTAGCTTTGCATTTGCTCATTGTATCA

TATCATTTTGCACCATTTTAGCTTGTTTTGCATACTGATATCTAGGATCATCTTGCATTC

GCATTGCATGTTTTGGTGTGTTTTAGGTGTAAAAGGAGCTTAAAAGGACTCTACTCGATC

CATTCACTCTATCCAGGCAGCTACAGAAGAGGCATCATTCTACTCGACCTCTTTACTCGA

TCCAGGATGCTATAAAGGAGGCTGCAGTTCACTCGACCCTCCTACTTGACCAAGGCAACC

TCTAATGAAGCAGCAGATTGATCTACTTGCCCTCCTACTCGATCCATTTACTCGACCAAG

GGTGACCATCTCTACAAGAGTTGTTGAGCTATCAAAGCGTCCTCGAATCCTCCTCATTCC

CCACTCAACTAGACATCTGAGCATACGGATATGAGAGGATGAAGCATCTACTCGACCAAT

TCATCACTCGGAGGATAGACACAAAGGCGTAGACTTTGTAGCACCCTCTTTACTCGATTC

CCAAGGTCGAGTATTCGCCCAAACCGCCTCAAGTCTCTTGTCTTCTACTGAATCCAGGGT

TTCAACATTTTAGTATAAATAGATCACTTTGGGATTTGTCCAAATGATCTATGCTTTATC

TTTCAGTTTTTCTTTGTGTTTATGAATCTTGAAACTTTAATTACTCTTAATCTATTTCGT

TTTTGCATTTGATTGTTTAAGTAAAGTTGTTTAGCTTGTTTAGATCTACATTTTGGTATG

ATTTTAGTAATTTCTCGGTTTGTGTTCTTGATAATGTTGCTTGAGTAGTGATTAGGGTTT

TTGAGGATGGATTAGATTAGGGTAATCTCTAAGTTAGTTAGGTGGTTAGTGTTTAGGTTT

ATACCCTTCTGGATTTGCTTGGCCCCTATGCGATTTTCGCTGGCTAATGTGGAGATTCGA

TCATAGACGTCCAGCGTCCAAAAGACGTTCAAATGGATGTCTGACCTACGAATTCTAGAG

ATACGTAGCCTCGACCAAGGGATTGGCTTGTCTGCCTATTGGCTTTGGTAGATTGTTTCT

TAAACCCTACTCTGCTATGTTTCATCAACGAGAGTTGAGATTAGGAAATGGTAGAGTTAT

GGGGGTCTCTGTCACGAGAGTGAATTGACCCGGTTTTTGAATCTATCGTCATGGGTATCT

ACCTTTCGCCTATTAGCCATTTGATGAACCCTAGAGTTGCCCTTATCAATGAATCCATCC

TTAGAACTTCTATTAGATTTATTTGTA
